# Acceptance of interventions to promote primary care: What do physicians prioritize?

**DOI:** 10.1186/s12875-015-0397-4

**Published:** 2015-12-15

**Authors:** Ryan Tandjung, Sima Djalali, Susann Hasler, Nathalie Scherz, Thomas Rosemann, Stefan Markun

**Affiliations:** Institute of Primary Care, University Hospital and University of Zurich, Pestalozzistrasse 24, 8091 Zurich, Switzerland

## Abstract

**Background:**

Switzerland is facing a shortage of primary care physicians (PCPs); government organizations therefore suggested a broad variety of interventions to promote primary care. The aim of the study was to prioritize these interventions according to the acceptance and perceived barriers of most relevant groups of physicians in this context (hospital physicians and PCPs).

**Methods:**

The study was conducted during summer 2014. An online-based questionnaire assessed demographic data, working conditions and future plans. Participants were asked to rank the usefulness of 22 interventions to promote primary care. Interventions to promote primary care that received ratings of 4 or 5 on the Likert scale (corresponding to “useful” or “very useful”) by at least 80 % of the participants were categorized as interventions with very high acceptance. We analyzed whether the groups (PCPs, hospital physicians) ranked the interventions differently using the Mann–Whitney *U* test. We assumed a two tailed *p* < 0.05 after Bonferroni correction for multiple testing as statistically significant.

**Results:**

Two hundred thirty physicians (response rate 58.4 %) completed the survey. Among those 69 PCPs and 66 hospital physicians were included in the analysis. Among those 14 PCPs were planning to leave clinical practice due to retirement, whereas only 8 hospital physicians planned a career as PCPs. Among PCPs the intervention with the highest acceptance was the increase of reimbursement, whereas family friendly measures achieved highest acceptance among hospital physicians. Financial support for primary care traineeships was considered to be very useful by both groups.

**Conclusions:**

Interventions on PCPs close to retirement or on PCPs considering an early retirement will not adequately prevent shortage of primary care providers. Governmental interventions should therefore also aim at encouraging hospital physicians to start a career in primary care by governmental support for traineeships in primary care and investments in family friendly measures.

## Background

As many industrialized countries, Switzerland is facing a shortage of primary care physicians (PCPs). In 2013, the average age of PCPs was 53.7 years [1] and a large proportion of PCPs will soon retire from clinical work. Meanwhile, medical students and young physicians do not perceive primary care as an attractive career choice [2, 3]. Seeing such indicators, concerns about future shortcomings in primary care arise, and a need for interventions to promote primary care is perceived. In order to counteract such a development the Swiss Federal Council has introduced a multifaceted intervention plan to strengthen primary care, aiming at better remuneration, new health services models and a better academic position [4]. The Swiss population has confirmed this direction in a public vote in 2014.

To achieve the goal of strengthening primary care, interventions must be selected and tailored to meet the needs of the targeted population of physicians. Without knowledge about necessity and acceptability of such interventions, the intended aims of promoting primary care could be missed. Also, in order to be economically sensible, interventions need to be delivered to specific groups or subgroups according to the potentially achievable results.

The aim of the study was to prioritize interventions to promote primary care according to acceptance by different groups of physicians. Furthermore these physicians’ views of barriers and interests towards a career in primary care were assessed in order evaluate the potential benefit of interventions in the respective groups. We were specifically interested to provide useful strategic information for policy makers.

## Methods

This is a cross-sectional survey among physicians working in the Swiss Canton of Zug (population in 2012: 116’575). The Canton of Zug represents a typical Swiss region, with an urban population living in towns and a typical hospital density. In the Canton, 196 physicians in outpatient care per 100’000 inhabitants were working (Swiss average: 210) [5]. The Canton has four inpatient institutions: Two hospitals for somatic diseases (one public, one privately funded), a public psychiatric clinic and a private rehabilitation clinic.

A questionnaire with overall 61 items was composed and entered in a web-based tool for surveys. A total of 405 e-mail addresses were eligible. These were obtained from the current and complete directories of the four hospitals and from the local physicians’ organization, where membership that includes registration with an e-mail address is mandatory for all physicians working in private practices. Thus, the eligible e-mail addresses has covered all physicians working in the Canton of Zug. A first invitation to participate in this study was sent on June 23^rd^ 2014. Participants who did not complete the survey after the initial invitation received reminding messages after three weeks and again on fifth week. There was no financial incentive for participation.

The questionnaire contained items on current working conditions, demographic data and participants’ future plans (hospital physicians: concerning future practice work; for physicians rejecting a career in outpatient care, the main barriers were assessed; and for physicians in outpatient care their intention to give up clinical work). Participants were asked to rate 22 predefined interventions on a 5-item-Likert scale regarding the usefulness of these interventions to promote primary care (1 = not useful at all up to 5 = very useful). The interventions were compiled by the local Health Department and included interventions that had been discussed in local and national politics. The interventions were categorized into six domains (1) reimbursement in primary care; (2) family-friendly measures; (3) investments in education and vocational training; (4) transition from hospital to practice; (5) regulatory measures and (6) eHealth.

We restricted our analyses to PCPs and hospital physicians at an early stage of their career. PCPs are the target group for interventions aiming to prevent PCPs quitting their work. Hospital physicians at an early stage of their career represent the target group to recruit new PCPs. Thus, chief physicians and attending physicians at hospitals and also specialist were excluded from the analysis, as interventions to promote primary care are hardly relevant to influence their career towards primary care. The remaining residents and senior physicians were grouped together and will hereinafter be referred to as “hospital physicians”.

### Statistics

We used descriptive statistics to analyze the results using counts and proportions for categorical data and means and standard deviations for continuous variables. Missing values were not imputed. Interventions to promote primary care that received ratings of 4 or 5 on the Likert scale (corresponding to “useful” or “very useful”) by at least 80 % of the participants were categorized as interventions with very high acceptance. We analyzed whether the groups (PCPs, hospital physicians) ranked the interventions differently using the Mann–Whitney *U* test. We assumed a two tailed *p* < 0.05 after Bonferroni correction for multiple testing as statistically significant. Statistical analysis was performed using R (version 3.1.0).

### Ethical issues and data confidentiality

During the period of data collection responsiveness of addressees was monitored and non-responders received reminders. Therefore, responses and address data of participants were linked. This link was deleted prior to the analysis of data in order to achieve complete anonymization. All data was treated confidentially. According to Swiss law [6], a survey among physicians does not require a vote of the Ethical Committee.

## Results

### Participation and characteristics of study groups

Eleven out of 405 eligible e-mail addresses were invalid; overall a total of 247 physicians logged into the platform and started the survey, 230 completed it. This corresponds to a response rate of 58.4 %. 139 (56.3 %) participants were working in outpatient care and 108 (43.7 %) in hospitals. Adhering to the analysis plan, participants working as specialists (*n* = 70) and working as attending or chief physicians in hospitals (*n* = 42) were excluded. The remaining 69 PCPs and 66 hospital physicians on an early career level were included for further analyses. Table 1 compares characteristics of these two groups of physicians.

**Table 1:** Data on age, sex and working conditions of the two groups in the focus of this survey

Variable	Category (description method)	Primary care physicians		Hospital physicians	
		*n* = 69		*n* = 66	
Sex	Female (n and %)	50	72.5 %	23	38.3 %
Age	(Mean and +/−SD)	54.0	+/−8.44	36.7	+/−7.48
Working days per week	less than 2 (n and %)	1	1.4 %	0	0.0 %
	2 to 3.5 (n and %)	11	15.9 %	5	7.6 %
	4 to 5.5 (n and %)	38	55.1 %	43	65.2 %
	more than 5.5 (n and %)	15	21.7 %	11	16.7 %
	information missing (n and %)	4	5.8 %	7	10.6 %

Working days were self-indicated by the participants in half-days. These were grouped into the categories

### Primary care physicians

Of the 69 PCPs 43 (62.3 %) were working self-employed, 13 (18.8 %) were working as employees and 13 (18.8 %) were employed by a corporation they were shareholders of. 42 (60.9 %) were working in a single-handed practice, 22 (31.9 %) in a group-practice and 5 (7.2 %) in a medical center. 14 (20.3 %) reported plans to give up their clinical work as PCPs within the next years, all but one of them because of regular age-related retirement. Mean age of PCPs was 54.0 years. Thus, PCPs were significantly older than hospital physicians (mean difference 17.3 years).

### Hospital physicians

Of the 66 hospital physicians 34 (51.5 %) were residents, 32 (48.5 %) were senior physicians. Eight (12.1 %) were considering working as a PCP in the future, 19 (28.8 %) working as specialist in outpatient care in the future. In participants rejecting a future work in a practice (*n* = 39, 59.1 %) the main barriers were assessed. The most common reasons chosen were: *I like to work in a bigger team* 30 (76.9 %); *regular exchange with colleagues is important to me* 26 (66.7 %), *I have chosen different career path, e.g. a hospital based specialty* 15 (38.5 %); and *I fear the high administrative work in practices* 15 (38.5 %).

**Fig. 1:**
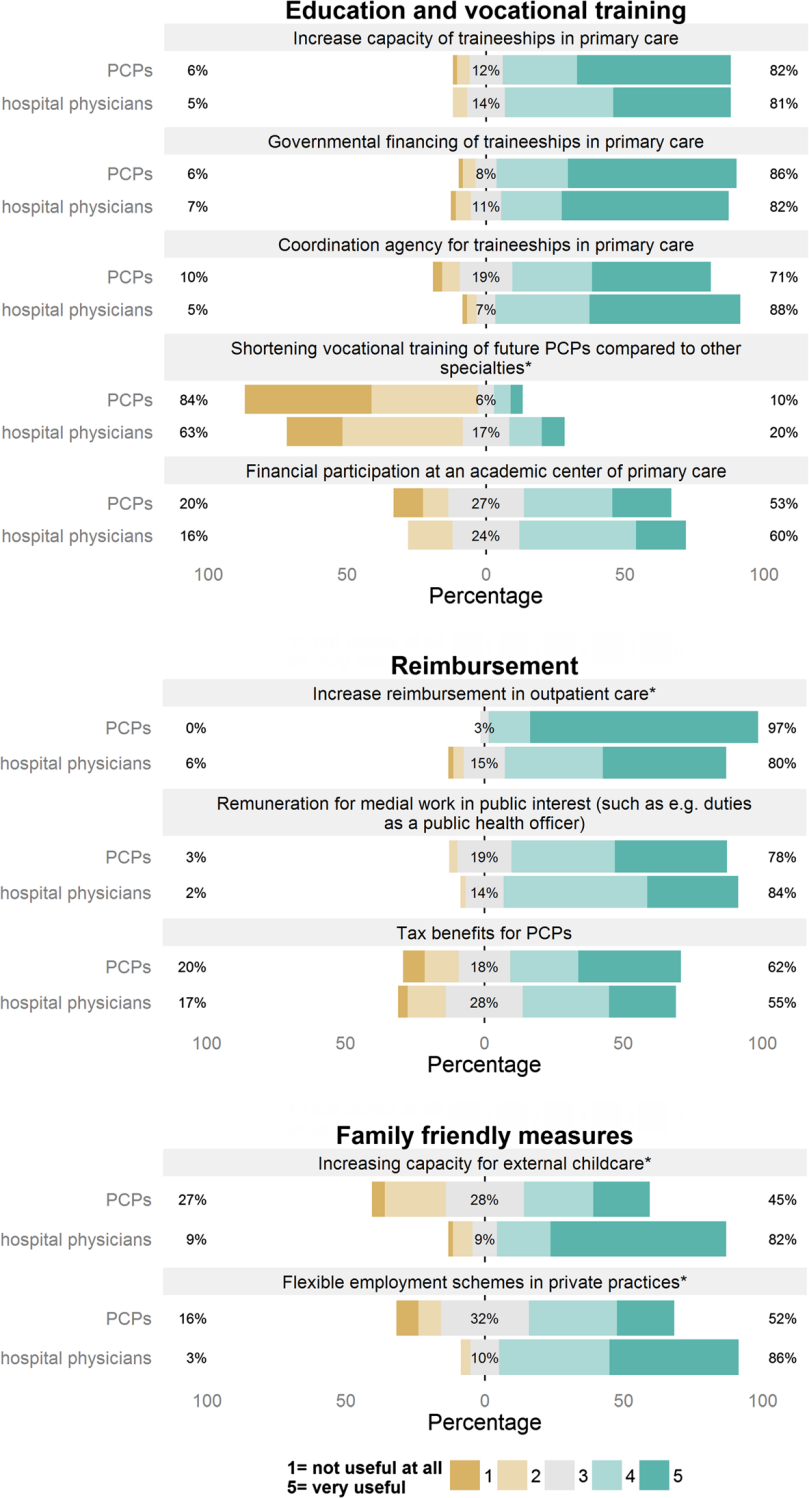
Shows how the hospital physicians and the primary care physicians (PCPs) rated the interventions to promote primary care for their usefulness on a 5-item-Likert scale (1 = not useful at all up to 5 = very useful). The interventions are grouped by the six domains and the physician groups, Fig. 1 shows three domains (education and vocational training, reimbursement, family friendly measures). The physicians’ ratings are displayed by horizontal stacked barplots, ratings indicating little acceptance (1 and 2) contribute to the left orange part of the bars, ratings indicating intermediate acceptance (3) contribute to the middle gray part and ratings indicating high acceptance the right green part, proportions of the parts are displayed in percentages. Interventions rated significantly different by the two groups of physicians are marked with an asterisk

**Fig. 2:**
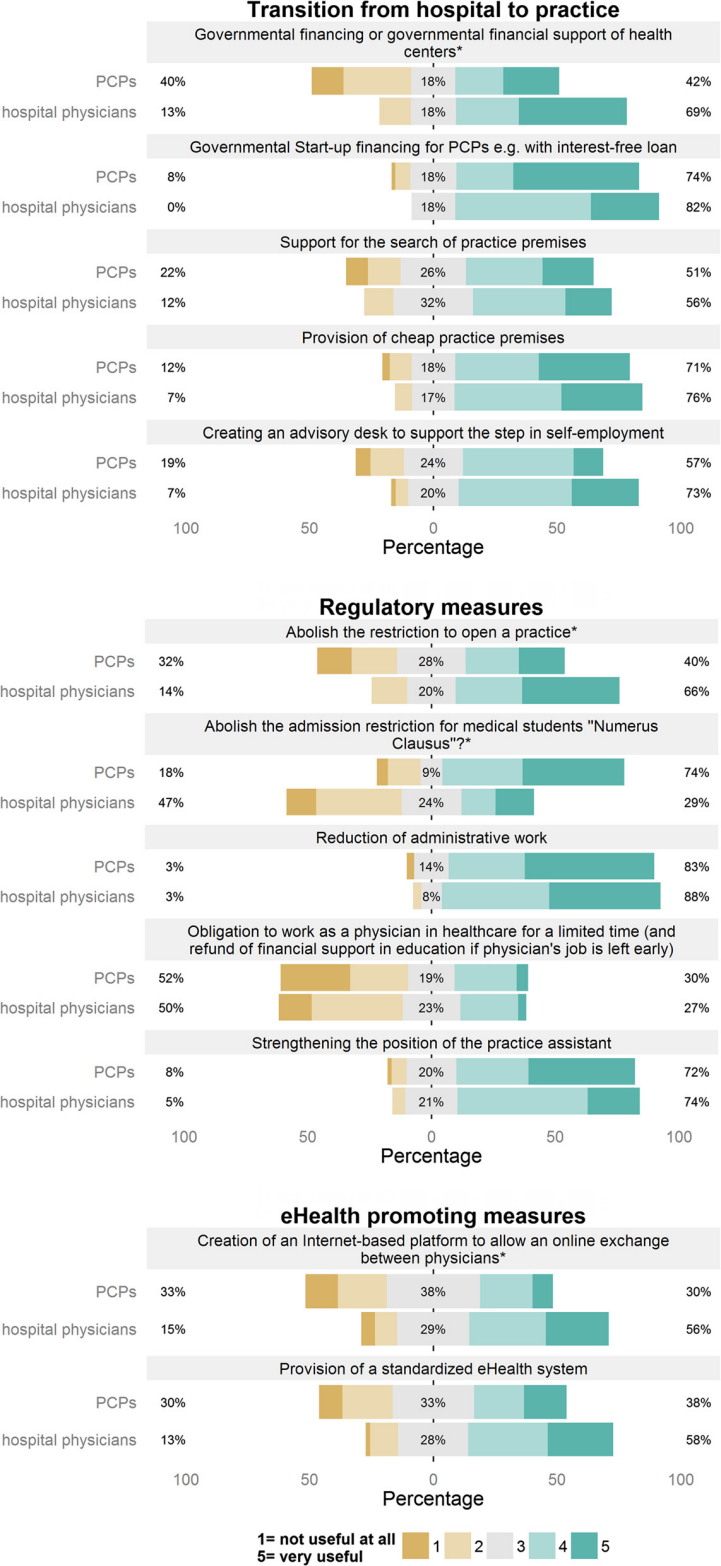
Shows further three domains (transition from hospital to practice, regulatory measures, eHealth promoting measures) how hospital physicians and primary care physicians (PCPs) rated the interventions to promote primary care for their usefulness on a 5-item-Likert scale (1 = not useful at all up to 5 = very useful). The physicians’ ratings are displayed by horizontal stacked barplots, ratings indicating little acceptance (1 and 2) contribute to the left orange part of the bars, ratings indicating intermediate acceptance (3) contribute to the middle gray part and ratings indicating high acceptance the right green part, proportions of the parts are displayed in percentages. Interventions rated significantly different by the two groups of physicians are marked with an asterisk

All participants were invited to rate 22 possible interventions for their usefulness to promote primary care. In overview, interventions in the reimbursement domain were rated as the most useful, whereas interventions in the domains of regulatory measures and eHealth were seen as the least useful. There was however, considerable variability between several interventions within the domains suggesting that the results should be examined on the level of the individual interventions rather than on the domain level. Acceptance was lowest in “*shortening of vocational training of future PCPs*” that was rated to be useful by less than 20 % of the participants and in “*obligation to work as a physician in healthcare for limited time (and refund of financial support in education if physician’s job is left early)*” that was rated to be useful by less than 30 %. In either group, interventions with very high acceptance (at least 80 % of the physicians rating them as useful) were “*increase capacity of traineeships in primary care*”, “*governmental financing of traineeships in primary care*”, “*increase reimbursement in primary care*” and “*reduction of administrative work*”. Some interventions achieved very high acceptance only in the group of the hospital physicians. These were “*coordination agency for traineeships in primary care*” (88 %), “*remuneration for medical work in public interest*” (84 %), “*increase capacity for external childcare*” (92 %), “*flexible employment schemes in private practices*” (86 %) and “*governmental start-up financing for PCPs*” (82 %). Figures 1 and 2 contain the detailed information about the usefulness ratings of all the interventions. Significant differences between the study groups were found in six out of the 22 items and are marked with an asterisk.

## Discussion

This is the first study investigating acceptance of a broad variety of interventions to promote primary care by different Swiss physician groups. The majority of interventions had similar acceptance from hospital physicians and PCPs, there were however important differences between those groups that need to be considered by initiatives strengthening primary care. Initiatives targeting current PCPs would encounter greatest acceptance when increasing reimbursement and strengthening traineeships in primary care. Initiatives targeting hospital physicians would encounter greatest acceptance when targeting at family friendly measures but also when strengthening traineeships in primary care.

In strategical view, the situation in the Canton of Zug showed that only 8 hospital physicians had the intention to become a PCP, while 14 PCPs were planning to give up clinical work. The number of PCPs will therefore further decline. Moreover, 13 out of the 14 PCPs will quit clinical work because of regular retirement; therefore, interventions targeting PCPs have little potential to prevent this decline no matter how welcome any intervention might be. Thus in short term, greatest potential to promote primary care lies in recruiting new physicians for the task. Unfortunately, however, we found that only a minority of hospital physicians is planning to become PCP. In our study, attitudes such as preferring teamwork and regular contact with peers were identified as barriers against a career in primary care, confirm findings of previous studies [2, 7–9]. The specific needs of hospital physicians are very important to address since they are the obvious resource to prevent a further increasing shortage of PCPs in the next years. This does not only apply to Switzerland, but to the majority of Western countries facing a comparable shortage of PCPs.

For hospital physicians, the family friendly measures and the traineeships in primary care were regarded as the most useful interventions. Consistently, earlier studies described physicians choosing primary care as career as more family orientated and more likely to have children [10, 11]. Therefore, flexibility to combine clinical work with the tasks of a young parent is a crucial factor for young, particularly female physicians’ career choice [12–15]. This finding underlines that promotion of primary care should not only focus on reimbursement, but also on the possibility to combine professional and private life [10, 16, 17]. Interestingly, family friendly measures were not as important to the physicians already working as PCPs, similar patterns could be found in other European countries [12, 18–20]. We believe that this finding is attributable to the different life situations of the studied groups. The younger hospital physicians are more concerned about compatibility of their private and professional circumstances than the older population of PCPs who have already run through the process of organizing family next to their work.

Also PCPs gave great importance to traineeships in primary care. Time spent in primary care is an important factor to increase motivation of medical students or young physicians to become a PCP [21, 22]. Studies indicate that interest for primary care is increases during residency; also residency was shown to be more determining for future career choice than medical school [3, 23, 24]. Additionally, in contrast to other countries, in Switzerland there is not a specific PCP track in vocational training but rather a common trunk for future general internists in practices as well as in hospitals [25]. Furthermore, the reported barriers against a career in primary care suggest that current mindsets about work in primary care might be based upon outdated stereotypes. We assume that enabling residents to experience how PCPs actually work nowadays might change these perspectives. Traineeships in primary are not only useful and effective interventions to facilitate the career pathway from hospitals to primary care but they are also very highly accepted among PCPs and hospital physicians.

Reduction of administrative work is an evergreen request of physicians and was popular as expected in both of our studied groups of physicians. Interventions targeting this issue are, however, very complex to design, require a multitude of involved contributors and might not be realistic to implement in a top-down approach. To enhance reimbursement in primary care is a similar request with obviously high acceptance. Still, inequalities between PCPs and specialists have been described in different health care systems and were found to be an important factor for career choices [26–29]. 80 % of the hospital physicians rated interventions increasing reimbursement in primary care to be useful but PCPs did so even in 97 %. This statistically significant difference might be explained by different life situations and furthermore because hospital physicians might actually be unaware of how remuneration is really generated in primary care. Regardless of this difference, increasing remuneration might be an effective incentive also for hospital physicians; still it was only ranked seventh in this population suggesting that other interventions could be prioritized. To consider income inequalities might however, be important to retain PCPs in their jobs on the long run because such inequalities have an impact on physicians’ satisfaction with their career choice and consecutively to the motivation of younger physicians to become PCPs [30, 31].

### Strengths and limitations

The response rate of 62.7 % was extraordinarily high for a survey with physicians [32]. The demographic data is comparable to data of the Swiss Medical Board [1]. We therefore claim our study sample to be representative. However, a few limitations need to be acknowledged. By definition, the catalogue of the 22 assessed interventions could not include all potentially existing ideas in this field. However, the catalogue was composed on the basis of the most often discussed political interventions. It must be acknowledged, that we could not completely avoid social desirability bias. Currently, the majority of health politicians seem to promote primary care. Thus, a physician might have felt urged to confirm the most common postulations instead of presenting his own opinions. Still, we consider the risk of this bias as being low, because the analysis of the groups showed that different groups did prioritize different interventions, indicating that the influence of public opinions is rather negligible.

## Conclusion

Reaching the retirement age is the predominant reason for PCPs to leave clinical work, governmental interventions aiming at retention of those PCPs are therefore less promising than interventions aiming at encouraging hospital physicians to start a career in primary care and close the opening gap. Governmental support of family friendly measures allowing young physicians to combine family planning and working as PCPs were best accepted by this target group. Traineeships in primary care, however, are also very highly esteemed by both groups of physicians and interventions should aim at ensuring attractiveness not only for the trainees but also for experienced PCPs who are indispensable to keep up such traineeships.
